# PADI-web corpus: Labeled textual data in animal health domain

**DOI:** 10.1016/j.dib.2018.12.063

**Published:** 2018-12-23

**Authors:** Julien Rabatel, Elena Arsevska, Mathieu Roche

**Affiliations:** a ASTRE, Cirad, INRA, Montpellier, France; b TETIS, Univ. of Montpellier, AgroParisTech, Cirad, CNRS, Irstea, Montpellier, France; c Cirad, Montpellier, France

## Abstract

Monitoring animal health worldwide, especially the early detection of outbreaks of emerging pathogens, is one of the means of preventing the introduction of infectious diseases in countries (Collier et al., 2008) [3]. In this context, we developed PADI-web, a Platform for Automated extraction of animal Disease Information from the Web (Arsevska et al., 2016, 2018). PADI-web is a text-mining tool that automatically detects, categorizes and extracts disease outbreak information from Web news articles. PADI-web currently monitors the Web for five emerging animal infectious diseases, i.e., African swine fever, avian influenza including highly pathogenic and low pathogenic avian influenza, foot-and-mouth disease, bluetongue, and Schmallenberg virus infection. PADI-web collects Web news articles in near-real time through RSS feeds. Currently, PADI-web collects disease information from Google News because of its international and multiple language coverage. We implemented machine learning techniques to identify the relevant disease information in texts (i.e., location and date of an outbreak, affected hosts, their numbers and clinical signs). In order to train the model for Information Extraction (IE) from news articles, a corpus in English has been manually labeled by domain experts. This labeled corpus (Rabatel et al., 2017) is presented in this data paper.

**Specifications table**

**Table:** 

Subject area	*Epidemiological surveillance in agriculture*
More specific subject area	*Text-mining approaches for animal health surveillance*
Type of data	*text file*
How data was acquired	*PADI-web crawler*
Data format	*JSON*
Experimental factors	*Evaluation of relevance (i.e. correct/incorrect) for each candidate entity*
Experimental features	*Textual entities with associated information such as spatial coordinates for locations*
Data source location	*Web*
Data accessibility	https://dataverse.cirad.fr/dataset.xhtml?persistentId=doi:10.18167/DVN1/KMTIFG

**Value of the data**

• *In Information Extraction (IE) domain*: This benchmark can be used to compare IE tools of the state-of-the-art for standard entities (e.g. locations, dates) and specific ones (e.g. diseases, hosts).
• *In Natural Language Processing (NLP) domain*: Disambiguation of locations based on spatial information (i.e. spatial coordinates).
• *In Information Retrieval (IR) domain*: As each document is labeled as relevant, related, or irrelevant, this dataset can be used for evaluating classification and/or clustering methods.
• *In visualization domain*: spatio-temporal visualization of data.
• *In epidemiology domain*: analysis of spatio-temporal information of exotic animal infectious diseases.

## 1 Data

In the context of epidemiological surveillance on the Web [3], this dataset [4] contains a set of news articles in English related to animal disease outbreaks, used to train and evaluate the information extraction module of the system PADI-web [2]. It is composed of 532 articles (in JSON) with information about the article itself (e.g., publication date, title, content, URL). When an article is evaluated as relevant by experts (i.e., the text describes a disease outbreak), the candidate entities of the articles are manually labeled. The candidates (e.g., locations, diseases, hosts, dates, etc.) - see Table 1 - have a *correct*, *partial*, or *incorrect* label, where *partial* is associated to candidates that, while they do not provide the exact needed information, are sufficiently similar to be of interest (e.g., a date that is close to the exact date of a disease outbreak).

**Table 1:** Summarization of labeled entities.

**Information**	**Label**	**Number of labeled candidates in the corpus**
Diseases	type = disease	921
Hosts	type = host	1139
Location	type = location info = {spatial coordinates}	4319
Date	type = date info = date value	994
Number of cases	type = number	1927

## 2 Experimental design, materials and methods

The dataset was constructed by collecting all notification reports sent to the World Organization for Animal Health (OIE) from 2014 to 2015 and available on their web page. Each report has been automatically processed to get the name of the disease, the country, the date of the outbreak and the date of the notification. For each report, a query has been built on Google News, to retrieve news articles which were published between the reported outbreak׳s starting date and the date of notification, and such that the title contained both the disease and the country name. For each query, the top ten news articles have been collected (or all articles when the query returned less than ten results). The queries resulted in 532 distinct news articles (HTML web pages) and were processed with Readability [https://www.readability.com] in order to extract the raw article content from the different Web pages.

Each article is labeled as *relevant* if it describes a disease outbreak, *related* if the disease outbreak is not the main topic of the article (e.g., an article that describes the economic impact of an outbreak) or *irrelevant* when the article has no connection to a disease outbreak.

In order to recognize candidate entities in texts for Information Extraction, our approach uses specific resources, tools, and methods:

– Specific dictionaries to identify diseases and hosts [1];
– GeoNames for location recognition [http://www.geonames.org];
– HeidelTime for date recognition [5];
– Regular expressions for identification of number of affected cases.

The candidates are highlighted in texts by using these different resources and tools. Each candidate entity from a relevant article has been manually labeled (i.e., *correct*/*partial*/*incorrect*) by two experts in epidemiology and health informatics (the first authors of this data paper). The labeled dataset was finally used to build a Support Vector Machine model in order to predict the relevance of each candidate in new documents. This model has been integrated into the PADI-web system. This dataset is an enriched version of the data that were used in PADI-web (the main difference being that partial candidate labels in PADI-web were all considered as correct) to obtain F-measure scores of 80% for locations, 83% for dates, 95% for diseases, 95% for hosts, and 85% for case numbers [2].
