# Supplementary material for: PADI-web corpus: Labeled textual data in animal health domain
Source: Data Brief. 2018 Dec 23;22:643–6. doi: 10.1016/j.dib.2018.12.063 (PMC6327737; doi:10.1016/j.dib.2018.12.063)
Supplement: Supplementary file 1 — Supplementary material [file mmc1.pdf]

Journal
Data in Brief

ID: DIB-D-17-01376R1

Montpellier, 20th December 2017

Conflict of Interest

Dear Editor,

We wish to confirm that there are no known conflicts of interest associated with this publication and there has been no significant financial support for this work that could have influenced its outcome.

We confirm that the manuscript has been read and approved by all named authors and that there are no other persons who satisfied the criteria for authorship but are not listed. We further confirm that the order of authors listed in the manuscript has been approved by all of us.

Yours sincerely,

Mathieu Roche, on behalf of all the authors
